# Genome-Wide Association Analyses Track Genomic Regions for Resistance to *Ascochyta rabiei* in Australian Chickpea Breeding Germplasm

**DOI:** 10.3389/fpls.2022.877266

**Published:** 2022-05-18

**Authors:** Rosy Raman, Annie Warren, Marzena Krysinska-Kaczmarek, Maheswaran Rohan, Niharika Sharma, Nicole Dron, Jenny Davidson, Kevin Moore, Kristy Hobson

**Affiliations:** ^1^NSW Department of Primary Industries, Wagga Wagga Agricultural Institute, Wagga Wagga, NSW, Australia; ^2^NSW Department of Primary Industries, Tamworth Agricultural Institute, Tamworth, NSW, Australia; ^3^South Australian Research and Development Institute, Urrbrae, SA, Australia; ^4^NSW Department of Primary Industries, Orange Agricultural Institute, Orange, NSW, Australia

**Keywords:** chickpea, Ascochyta blight, DArTseq-SNP, GWAS, QTL

## Abstract

Ascochyta blight (AB), caused by a necrotrophic fungus, *Ascochyta rabiei* (syn. *Phoma rabiei*) has the potential to destroy the chickpea industry worldwide, due to limited sources of genetic resistance in the cultivated gene pool, high evolutionary potential of the pathogen and challenges with integrated disease management. Therefore, the deployment of stable genetic resistance in new cultivars could provide an effective disease control strategy. To investigate the genetic basis of AB resistance, genotyping-by-sequencing based DArTseq-single nucleotide polymorphism (SNP) marker data along with phenotypic data of 251 advanced breeding lines and chickpea cultivars were used to perform genome-wide association (GWAS) analysis. Host resistance was evaluated seven weeks after sowing using two highly aggressive single spore isolates (F17191-1 and TR9571) of *A. rabiei.* GWAS analyses based on single-locus and multi-locus mixed models and haplotyping trend regression identified twenty-six genomic regions on Ca1, Ca4, and Ca6 that showed significant association with resistance to AB. Two haplotype blocks (HB) on chromosome Ca1; HB5 (992178–1108145 bp), and HB8 (1886221–1976301 bp) were associated with resistance against both isolates. Nine HB on the chromosome, Ca4, spanning a large genomic region (14.9–56.6 Mbp) were also associated with resistance, confirming the role of this chromosome in providing resistance to AB. Furthermore, trait-marker associations in two F_3_ derived populations for resistance to TR9571 isolate at the seedling stage under glasshouse conditions were also validated. Eighty-nine significantly associated SNPs were located within candidate genes, including genes encoding for serine/threonine-protein kinase, Myb protein, quinone oxidoreductase, and calmodulin-binding protein all of which are implicated in disease resistance. Taken together, this study identifies valuable sources of genetic resistance, SNP markers and candidate genes underlying genomic regions associated with AB resistance which may enable chickpea breeding programs to make genetic gains *via* marker-assisted/genomic selection strategies.

## Introduction

Chickpea (*Cicer arietinum* L.) is an annual legume of the family Fabaceae and provides a healthy source of protein, carbohydrates with a low glycemic index, vitamins, and minerals for human consumption. Chickpea also provides rotational benefit at a whole-farm level, due to its ability to fix atmospheric nitrogen and break the cycles of pests, disease and weeds. However, its production is constrained by several abiotic and biotic stress factors, especially diseases. Ascochyta blight (AB), caused by a necrotrophic fungus, *Ascochyta rabiei* (syn. *Phoma rabiei*) is a major endemic disease of chickpea worldwide. In Australia, this disease causes an average of $4.8 million loss to the chickpea industry annually and without the current control measures, the losses are estimated to be $39.7 million (Murray, 2012). The AB pathogen can infect all aerial parts of the plant, resulting in girdling and breakage of the main stem and branches, the primary determinants of seed yield (Sharma and Ghosh, 2016). *Ascochyta rabiei* is seed-borne and survives in the infected stubble and on volunteer plants, which become the primary sources of infection in subsequent seasons. Under ideal temperatures of 15–25°C, leaf wetness and high humidity, the pathogen can cause widespread and severe infection within a short time (Kaiser et al., 2000).

Multiple management strategies based on crop rotation, stubble management, and fungicide applications are employed by growers to manage the disease in commercial chickpea crops, in combination with the genetic resistance of current cultivars. Deployment of stable genetic resistance in cultivars is recognized as one of the defensive strategies to control yield losses caused by the pathogen. Genetic variation for resistance to AB exists in cultivated, landrace accessions of *C. arietinum* and related wild *Cicer* species (Sharma and Ghosh, 2016). However, none of the resistant sources is completely immune to *A. rabiei* pathotypes. Since the first recorded *A. rabiei* epidemic in Australia in 1998, extensive efforts have been made to develop resistant cultivars (Du et al., 2012). The Australian chickpea breeding program (PBA, pulse breeding Australia; subsequently CBA, chickpea breeding Australia) has extensively deployed genetic resistance derived from an Iranian landrace, ICC3996 in several commercial chickpea cultivars (Li et al., 2017). However, due to the evolution of new pathotypes, this source has become less effective in conferring resistance to AB (Moore et al., 2015, 2016; Newman et al., 2020; Ford et al., 2021). Consequently, most of the commercial cultivars with resistant (R)/moderately resistant (MR) ratings have become moderately susceptible (MS)/susceptible (S). For example, Australian resistant kabuli (Genesis090) and desi (PBA Seamer) cultivars and widely used resistant landrace accession, ICC3996 have become moderately susceptible/susceptible to highly pathogenic isolates of *A. rabiei* under field environment (Mehmood et al., 2017; Ford et al., 2021). Similar findings have been reported in worldwide chickpea cultivars due to variation in the aggressiveness of the global *A. rabiei* populations (Vail and Banniza, 2008; Deokar et al., 2019b). It has been widely recognized that limited variation in genetic resistance and erosion of genetic resistance due to shifts in the aggressiveness of pathogen populations are major impediments to the development of cultivars with durable resistance. To address these challenges, breeders seek novel sources of resistance for developing improved cultivars with durable resistance with specific/broad adaptation depending on the target growing environment. Therefore, detailed knowledge of the genetic mechanism underlying host-pathogen interaction is essential to minimize yield losses due to AB.

Genetic analysis studies using bi-parental populations have revealed that AB resistance is controlled by multiple genes. To date, more than 80 quantitative trait loci (QTL) for resistance have been identified on Ca1 (Flandez-Galvez et al., 2003; Daba et al., 2016; Deokar et al., 2019a), Ca2 (Cho et al., 2004; Anbessa et al., 2009; Madrid et al., 2014; Deokar et al., 2019a,b), Ca3 (Flandez-Galvez et al., 2003; Aryamanesh et al., 2010; Daba et al., 2016), Ca4 (Stephens et al., 2014; Daba et al., 2016; Deokar et al., 2019a; Sudheesh et al., 2021), Ca5 (Sabbavarapu et al., 2013; Deokar et al., 2019b), C6 (Sabbavarapu et al., 2013; Deokar et al., 2019a,b), Ca7 (Daba et al., 2016; Deokar et al., 2019a), and Ca8 (Anbessa et al., 2009; Daba et al., 2016) under glasshouse and field conditions. These QTL accounted for 12–50% of the phenotypic variation, suggesting small to moderate allelic effects. Using *Fst* based genome–scan and GWAS approach, Li et al. (2017) identified a 100 kb region (AB4.1) for resistance to AB on Ca4 in Australian breeding germplasm. However, the relevance of described QTL in current Australian breeding germplasm against highly aggressive *A. rabiei* isolates is not known.

Chickpea Breeding Australia (CBA) has been incorporating genetic resistance from several diverse sources for the last two decades and developing improved varieties for growers. This set of breeding germplasm provides an excellent opportunity to identify alleles/loci for traits of interest, including AB resistance, that have accumulated as a result of recombination and selection under diverse pathogen populations. In addition, breeding populations offer several advantages; any QTL identified will be of direct relevance for the resistance breeding pipeline (Würschum, 2012). Also, any known AB loci/alleles not present in the breeding germplasm can be targeted to develop a knowledge-based approach to incorporate favorable loci into the breeding gene pool.

In this study, we investigated (i) the extent of genetic variation for AB resistance in the advanced Australian breeding germplasm against highly aggressive isolates and (ii) identified SNPs/haplotypes and underlying putative candidate genes associated with resistance at the adult plant stage. Utilizing 251 advanced breeding lines, phenotypic, and genotyping-by-sequencing based DArTseq-SNP marker data, we identified 26 genomic regions for resistance to two highly aggressive isolates of *A. rabiei*. Of them, two genomic regions on Ca1: HB5 (992178–1108145 bp) and HB8 (1886221–1976301 bp) were detected with both isolates. We also validated genomic loci on chromosome Ca1, associated with resistance to TR9571 isolate in two F_3_ populations derived from crosses involving moderately resistant breeding line, CICA1841. The SNPs/haplotypes identified herein will provide chickpea breeders and prebreeders the genotypes with favorable haplotypes as parents and further accumulate diverse and novel alleles from domestic and wild accessions using genomic selection strategies to develop cultivars with an optimum and stable level of resistance against the most damaging necrotrophic fungus.

## Materials and Methods

### Plant Materials

A GWAS set (*n* = 251) of advanced Australian breeding germplasm; comprising 227 lines from stage 2 (S2) and stage 3 (S3) of the breeding cycle, 20 commercial cultivars, three FLIP (Food Legume Improvement Program) lines and one AB resistant Iranian landrace (ICC3996) was selected for this study (Supplementary Table 1). Stage 2 and 3 refer to the multi-tier evaluation and selection system of elite lines within the CBA. From S3, elite lines are submitted to the National Variety Trials (NVT) for independent evaluation, before a line is released as a variety for commercial production. Two breeding F_3_ populations derived from PBA Drummond (highly susceptible to AB)/CICA1841 (moderately resistant to AB) and CBA Captain (susceptible to AB)/CICA1841 (moderately resistant to AB) crosses, were selected to verify QTL associated with resistance. All three parents of the F3 population were included in the GWAS set. Seed of all genotypes was obtained from New South Wales Department of Primary Industries (CBA), Tamworth, Australia.

### Phenotypic Evaluation of Genome-Wide Association Set

Genome-wide association set of 251 genotypes was evaluated for AB resistance in two independent experiments with the two highly aggressive isolates of *A. rabiei* (Supplementary Table 1). All experiments were conducted in a shade-house at the South Australian Research and Development Institute (SARDI), Adelaide, Australia (34.9670°S, 138.6360°E), maintained under ambient conditions with supplementary irrigation and overhead misting to enhance pathogen infection and disease development. Due to space constraints, the GWAS set was assessed for resistance to AB in two sets; 149 genotypes (130 S3 breeding lines, 18 cultivars and one AB resistant landrace, ICC3996) and 100 genotypes (96 S2 breeding lines, one *Cicer echinospermum* interspecific breeding line and three FLIP lines). Two Australian chickpea commercial cultivars; Howzat (highly susceptible) and Genesis 090 (moderately resistant) were included as “reference checks” in all experiments (Supplementary Table 1).

For all experiments, four plants of each accession were grown in pots (17.5 cm diameter) in a randomized complete block design with three replicates. The two highly aggressive isolates; F17191-1 (collected from the southern region, Port Broughton, SA, Australia) and TR9571 (collected from the northern region, Gurley, NSW, Australia) were used to assess disease response and identify genomic regions associated with resistance to these isolates. Both isolates belong to the same pathotype group (Group 5) but genetically relate to different clusters; TR9571 (Cluster E), F17191-1 (Cluster C) (Bar et al., 2021).

Seven-week-old seedlings (with an average of six fully developed nodes) were separately inoculated with *A. rabiei* isolate, F17191-1 (Experiment 1) or TR9571 (Experiment 2). Plants were spray inoculated to runoff at a concentration of 1 × 10^6^ pycnidiospores mL^–1^. After inoculation, the plants were maintained under high humidity with overhead misting, applied at a rate of 30 s every half an hour. Plants were watered using sprinklers as required until disease assessment. At 48 days after inoculation, host-pathogen interaction response was recorded at the adult plant stage on a pot basis (genotype) assessing the fraction of main stems broken, the fraction of the main stems with lesions, the fraction of side branches with lesions and the fraction of leaf area diseased. These data were converted to score of 1–9 as follows: 1 = no disease; 2 = no stem infection, 2% foliage infected; 3 = <5% stems broken, 30% main stems lesions, 10% foliage infected; 4 = 15% main stems broken, 60% main stem lesions, 25% minor stems lesions, 20% foliage infected; 5 = 40% main stems broken, 80% main stem lesions, 65% minor stems lesions, 50% leaves infected; 6 = 50% main stems broken, 100% stem lesions, 85% minor stems lesions, 60% leaves infected; 7 = 75% main stems broken, 100% stem lesions, 96% minor stem lesions, 75% leaves; 8 = 100% main stems broken, 100% stem lesions, 100% minor stems lesions, 90% leaves infected, and 9 = plant dead (100% infection).

### Phenotypic Evaluation of F_3_ Populations

Phenotypic assessment of two F_3_ populations; PBA Drummond/CICA1841 (240 lines) and CBA Captain/CICA1841 (240 lines), including parents (CICA1841, CBA Captain and PBA Drummond), four check cultivars (Kyabra, Genesis090, PBA HatTrick, PBA Seamer), a Syrian landrace, ICC3996, and twelve breeding lines was performed under glasshouse conditions at Tamworth, Australia (Latitude: −31.1333 Longitude: 150.9500) using the TR9571 isolate. The 10-day old seedlings were inoculated using 5 × 10^5^ pycnidiospores mL^–1^ and were maintained under high humidity with misters for 24 h following inoculation, at a rate of one minute every half an hour. After which, the misting was set for three minutes once per day until disease assessment. Disease infection was scored 14 days after inoculation using a modified 1–9 disease rating scale based on the disease reaction of an individual plant at the seedling stage. The 1–9 disease score is described as follows: 1 = no disease; 2 = few leaf lesions – small, 3 = few leaf lesions – large; 4 = 3 or less infected whole leaves, no stem lesions; 5 = more than 3 infected whole leaves, with small stem lesions (<1 cm); 6 = more than three infected whole leaves, with large stem lesions (>1 cm); 7 = numerous large stem lesions, but still some green leaves and growing point dead; 8 = leaves dead, stem green, growing point dead; and 9 = plant dead, no green tissue.

### Statistical Analyses

All statistical analyses were conducted with R version 4.0.5 statistical software (R Core Team, 2021). The package ASReml – R version 3 was used to perform the linear mixed effect model for estimating the predicted mean of disease score for genotypes in the GWAS set. A Shapiro-Wilk test was used to examine the data for normality. The best suitable model was selected after performing the likelihood ratio test for random effects and the Wald test for fixed effects. In the model, both column and row, representing the experimental unit, were included as fixed effects, and test-lines and replicates were considered as random effects plus the error structure of first-order autoregressive [AR (1)] was added to the model to accommodate the spatial correlation in both directions of columns and rows. The predicted means of the disease score for both isolates are depicted in the histogram and scatter plots.

The generalized linear mixed model with logit link of the binomial family was applied to satisfy the disease measure scale (fraction) for main stem broken (MSB), branch diseased (BD) and stem lesions (SL). The predicted mean of four disease measures against two isolates is depicted as box plots. Correlation coefficients for eight variables were calculated to evaluate the association among four disease measures with two isolates.

Broad-sense heritability (*H*^2^) of all measures of AB was calculated using the following equation: $H^{2}=1-\frac{\bar{v}}{2\times \sigma_{g}^{2}}$, where $\sigma_{g}^{2}$ refers to variance of genotype, and $\bar{v}$ is the average standard error of the predicted means (Cullis et al., 2006). In addition, the Pearson correlations were calculated to measure the degree of relationship between each disease measure for each individual isolate and across both isolates.

### Genotyping of Genome-Wide Association Set and F_3_ Populations

Genomic DNA was extracted from 10-day old seedlings as described previously (Raman et al., 2005). All germplasm accessions were genotyped using the genotyping-by sequencing-based DArTseq platform as described in Raman et al. (2014). The DArTseq method generates two types of polymorphisms: (i) *in silico* DArT (presence/absence markers) and (ii) DArTseq SNP (codominant). In this study, only DArTseq SNP markers were used for GWAS analyses. A total of 3,918 DArTseq SNP markers were polymorphic in the GWAS set. A sub-set of 2,130 high-quality DArTseq-SNP markers with a high call rate (>85%) and minor allele frequency (>2%) were further utilized for GWAS. Six kompetitive allele-specific PCR (KASP) markers associated with AB QTL on Ca2 and Ca4 reported by Deokar et al. (2019a) were also used to genotype the GWAS set and were included in the genetic analysis (Supplementary Table 2). DArTseq marker sequences were aligned with kabuli reference genome; Frontier version 2.6.3^1^ to predict the physical position of each marker.

The F_3_ population derived from PBA Drummond/CICA1841 (165 lines) and both parents were genotyped with DArTseq markers as described above. For CBA Captain/CICA1841 F_3_ population (224 lines), a subset of 45 moderately resistant (AB score 4–5) and 45 susceptible (AB score = 7–8) including parents, were genotyped using DArTseq markers. Marker data filtering was applied as described above for the GWAS set and 390 and 576 DArTseq-SNP markers generated from PBA Drummond/CICA1841 and CBA Captain/CICA1841 populations, respectively were used for QTL analyses.

### Population Structure Analysis

DArTseq markers were used to generate a phylogenetic tree in MEGA (Kumar S. et al., 2018). We performed principal component analysis (PCA) (Price et al., 2006) implemented in the SNP and Variation Suite (v8.1.5, Golden Helix, Inc., Bozeman, MT, United States).^2^ A plot of the first two principal components was created to visualize the possible population structure of the GWAS set. A kinship matrix to control for the relatedness among chickpea cultivars/breeding lines was computed from the identity-by-state distances matrix as executed in the SVS package Version8.6.0 (Golden Helix, Inc., Bozeman, MT, United States; see text footnote 2). The extent of linkage disequilibrium (LD) based on adjacent pair-wise ***r***^2^ values between high-quality SNPs from the GWAS set and physical distances between these SNPs was estimated using the Synbreed package in R (Wimmer et al., 2012). Non-linear models were fitted into the genome-wide and chromosome-wise LD data using the “nlin” function in R with ***r***^2^ as responses (***y***) and pair-wise physical distances (*Mbp*) as predictor variables as described by R Core Team (2021). The LD decay (***r***^2^ = 0.2) was measured using the stochastic gamma model with inverse link (Rohan et al., 2015). Genome-wide, and for each chromosome (Ca1–Ca8) LD decay plots, were plotted in R package GGPLOT2 for visualization.

### Genome-Wide Association Analyses

To identify genome-wide associations, both population structure (PCA; fixed effects) and kinship matrix (random effects) were accounted for in this study. GWAS was conducted using efficient mixed-model association expedited (EMMAX) single-locus (SLMM) and multi-locus mixed model (MLMM) in SVS V_8.6.0 software to identify the association between AB resistance (measured as disease score) and SNP markers (Kang et al., 2010; Segura et al., 2012). MLMM uses both forward and backward stepwise approaches to select markers as fixed effect covariates as developed by Segura et al. (2012). The critical threshold of significance was −log_10_ (*p*-value) ≥ 5.0, and Bonferroni *P*-value cut-off ≤ 0.01. In addition, haplotype trend regression (HTR) was performed which takes one or more block(s) of genotypic markers and for each block of markers, estimates the haplotypes for these markers, then regresses their by-sample haplotype probabilities against a dependent variable (Golden Helix, Inc., Bozeman, MT, United States; see text footnote 2). Haplotypes block and HTR analyses were performed using an algorithm implemented in SVS v8.1.5. HB were constructed for Ca1 to Ca8 chromosomes using SNPs with a MAF ≥ 0.05, ninety-eight (98%) percent upper confidence intervals of the “*D*” values and the lower boundary to 0.70. Haplotype frequencies were estimated using the Expectation Maximization (EM) algorithm with a frequency threshold of 0.01, EM convergence tolerance (0.0001) and 1,000 iterations. HTR analysis was performed based on stepwise regression with forward elimination, pre-computed HB as described above, and the first three principal components as fixed covariates. False Discovery Rate (FDR) and Bonferroni corrected *P*-values were imputed, and significant HB were called based on Bonferroni and FDR *P*-value cut-off ≤0.01 and ≤0.001, respectively. Linear marker regression was performed to verify QTL associated with AB resistance in two F_3_ populations derived from PBA Drummond/CICA1841 (390 SNP) and CBA Captain/CICA1841 (576 SNP) using SVS package. Furthermore, the PC (Principal component) of the GWAS set and each F_3_ population were estimated using common set of DArTseq SNP markers in the SVS package to gauge the relatedness between them. The first two PC, explaining most of genotypic variance were plotted to show clustering of GWAS and F_3_ populations.

Genomic regions associated with AB resistance were designated as chromosome name followed by the physical location (bp) of the DArTseq SNP based on kabuli reference genome (v 2.6.3) to aid in QTL comparison in this study and across previous studies. Manhattan plots were generated using the −log_10_ *P* values of all SNPs. QQ plots of the observed −*log_10_ P* values (Y-axis) and the expected −log_10_ *P* (X-axis) were plotted to check for genomic inflation.

### Comparison of Quantitative Trait Loci With Previous Studies and Candidate Gene Analysis

To compare the genomic regions identified in this study with the previously reported AB QTL, the information provided by Deokar et al. (2019b) was updated (Supplementary Table 6). The physical position of the QTL regions on the CDC Frontier genome assembly v2.6.3 (see text footnote 1) was determined using the SNP/SSR marker sequences as described by Deokar et al. (2019b). We searched putative candidate genes having annotations in reference assembly that map within 20 kb upstream and downstream regions of significant SNP associations identified in this study. The DarTseq marker sequences were searched against the chickpea genome assembly v 2.6.3 using BLASTn with word size 7 and e-value < 1e-10.

## Results

### Phenotypic Evaluation of Australian Chickpea Breeding Germplasm With Two Aggressive *Ascochyta rabiei* Isolates

**FIGURE 1:**
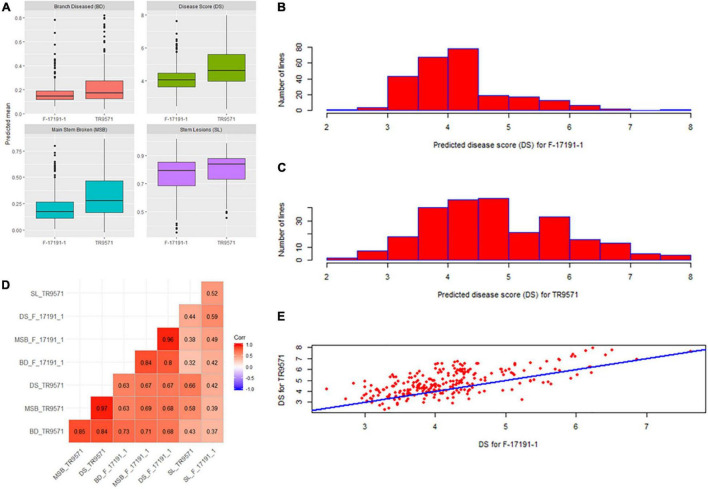
Genetic variation for resistance to Ascochyta blight in a genome-wide association (GWAS) set. **(A)** Box plot of four disease measures against two *Ascochyta rabiei* isolates. **(B)** The frequency distribution of disease scores in the GWAS set against F-17191-1 isolate. **(C)** The frequency distribution of disease scores in the GWAS set against TR9571 isolate. **(D)** Pair-wise correlations between different disease measures scored using two isolates. **(E)** Scatter plot showing relationship between predicted disease scores in the GWAS set after infection with F17191-1 and TR9571 isolates.

The disease response of the GWAS set was evaluated against two highly aggressive isolates of *A. rabiei*, TR9571 and F17191-1, using four measures of disease assessment: disease score, main stem breakages, stem lesions and branches diseased. Most disease measures had high heritability values, ranging from 52 to 82% (Table 1). Phenotypic analyses revealed significant variation for resistance to both isolates (Table 2 and Figure 1A). The disease score ranged from 2.46 to 7.63 among GWAS genotypes, while two check cultivars, Genesis090 (moderately resistant) and Howzat (susceptible) had a disease score of 3.3 and 7.3 (TR9571) and 3.1 and 6.4 (F17191-1), respectively. The AB resistant landrace, ICC3996 had a disease score of 4.9 and 3.77 with TR9571 and F17191-1, respectively (Supplementary Table 1). None of the genotypes were completely immune to AB infection, as they all had stem lesions and infected branches. The continuous variation in disease scores was observed among GWAS accession in response to both isolates, indicating that the AB resistance is quantitative in nature (Figures 1B,C).

**TABLE 1:** Summary of the statistical analysis of genome-wide association (GWAS) set of Australian breeding germplasm evaluated against two *Ascochyta rabiei* isolates.

Experiment	Isolate	Disease measure	Scale	Mean	Minimum	Maximum	Median	Standard deviation	Broad sense heritability (*H*^2^) (%)
GWAS set	F17191-1	Disease Score	1–9	4.2	2.46	7.63	4.06	0.83	74
		Main stem broken (MSB)	0-1	0.21	0.01	0.8	0.17	0.16	78
		Stem lesions (SL)	0-1	0.76	0.35	1.02	0.79	0.14	62
		Branches diseased (BD)	0-1	0.18	0.06	0.79	0.15	0.1	77
GWAS set	TR9571	Disease Score	1–9	4.79	2.29	7.97	4.64	1.13	79
		Main stem broken (MSB)	0-1	0.32	-0.03	0.87	0.28	0.2	82
		Stem lesions (SL)	0-1	0.8	0.46	0.99	0.84	0.11	52
		Branches diseased (BD)	0-1	0.23	0.04	0.82	0.17	0.15	78

**TABLE 2:** Summary of genetic loci associated with Ascochyta blight resistance in GWAS set of Australian chickpea breeding germplasm against two *Ascochyta rabiei* isolates using DArTseq SNP-based SLMM and MLMM.

Isolate	Model	Chr	DArTseq-SNP Marker	Physical Position (bp)	-log_10_ (*P*-Value)	Bonferroni *P* Value	FDR	PV (%)	Gene ID	Start (bp)	End (bp)	Annotation	Distance from SNP (bp)
F17191-1	MLMM	Ca1	8776174| F| 0-13:T > C	1820681	6.38	8.88E-04	4.44E-04	10.33	Ca07004	1817615	1822820	Putative PAP-specific phosphatase	0
	MLMM	Ca4	8776003| F| 0-61:T > C	36231638	6.70	4.28E-04	4.28E-04	9.79	Ca13516	36225721	36232356	DNA repair protein	0
	SLMM	Ca4	8776003| F| 0-61:T > C	36231638	6.50	6.81E-04	6.81E-04	10.14	Ca13516	36225721	36232356	DNA repair protein	0
	SLMM	Ca4	8822799| F| 0-51:G > C	36231694	5.52	6.48E-03	3.24E-03	8.53	Ca13516	36225721	36232356	DNA repair protein	0
	SLMM	Ca4	8776026| F| 0-55:A > G	36218164	5.51	6.64E-03	2.21E-03	8.51	Ca13513	36217006	36219504	F-box protein	0
TR9571	MLMM	Ca1	10270127| F| 0-58:A > T	1781336	5.78	3.56E-03	1.78E-03	8.94	Ca06994	1785328	1789751	Lysine-specific demethylase	3,992
	MLMM	Ca1	10265685| F| 0-59:G > A	7285403	5.18	1.42E-02	3.54E-03	7.83	Ca07687	7283110	7290006	Probable ADP-ribosylation factor GTPase-activating protein	0
	MLMM	Ca4	10266440| F| 0-5:C > T	15657995	6.20	1.34E-03	1.34E-03	9.53	Ca12147	15654405	15658065	Regulator of Vps4 activity in the MVB pathway protein	0
	MLMM	Ca4	8776114| F| 0-21:C > T	36327109	5.51	6.64E-03	2.21E-03	8.37	Ca13522	36326928	36333894	Exostosin family protein	0

*MLMM, multi-locus mixed model; SLMM, single-locus mixed model; FDR, false discovery rate; PV, percentage of phenotypic variation; Physical position is based on Kabuli Reference genome; Frontier v2.6.3.*

We observed moderate to high correlations between different measures of AB assessment. For example, disease score (1–9) had higher correlations with MSB and BD but the same had low correlations with SL (Figure 1D). A moderately high correlation (73%) between disease scores with two isolates indicate common as well as different genomic regions associated with AB resistance in the GWAS set (Figure 1E).

### Genotyping Using DArTseq SNP and Kompetitive Allele-Specific PCR (KASP) Markers

**FIGURE 2:**
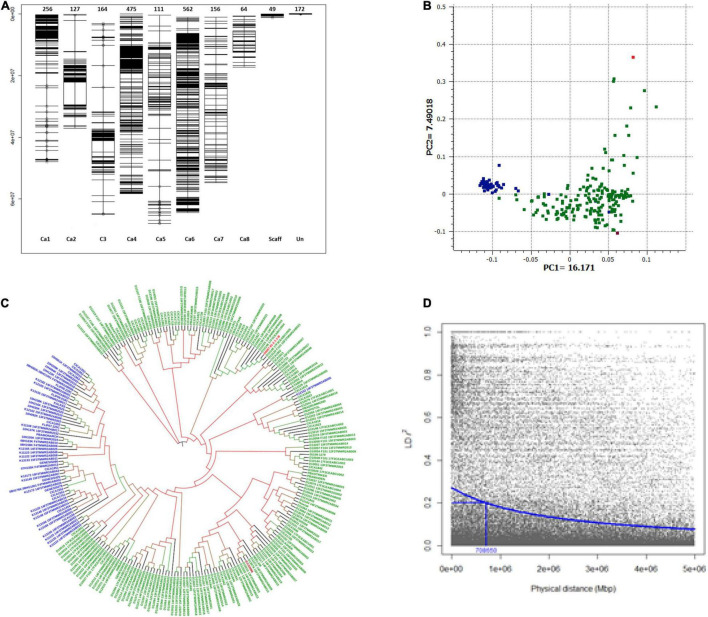
Population structure and linkage disequilibrium (LD) decay plot of GWAS set of Australian chickpea genotypes. **(A)** Distribution and position of SNP markers on chickpea genome used to genotype GWAS set of 251 chickpea genotypes. Chromosomes: Ca1 to Ca8, Scaff: Scaffold, Un: Unlinked markers. **(B)** Principal component plot: A scatterplot of first two PCs corresponding to 251 desi and kabuli chickpea genotypes, blue: kabuli genotypes, green: desi genotypes, red: *C. echinospermum* interspecific line, maroon: landrace (ICC3996); **(C)** Neighbor-joining phylogenetic tree. **(D)** LD decay plot of all eight chickpea chromosomes. The squared correlation coefficient *r*^2^ values (Y-axis) were plotted against the physical distance in megabase pairs (Mbp) (X-axis) using R Package Synbreed. LD decay (*r*^2^ = 0.2) occurs at approximately 0.70 Mbp.

A total of 2,130 high-quality DArTseq SNP and six KASP markers were selected for genetic analyses; of which 1,915 SNPs could be anchored on all eight chromosomes (Ca1–Ca8) of the reference chickpea genome assembly (Figure 2A). Anchored markers covered the physical distance of 414.79 Mbp. Chromosome Ca6 had the maximum density (562 SNP markers) while the Ca8 had the least marker density (64). Forty-nine (49) markers were mapped on unanchored scaffolds, whereas 172 could not be mapped on the reference genome.

### Population Structure and Linkage Disequilibrium

Principal component analysis differentiated the majority of the GWAS accessions into three groups. The first two principal components explained 23.66% of the genotypic variation (Eigen vector; EV1 = 16.71%, EV2 = 7.49% and separated kabuli and desi genotypes into two groups (Figure 2B). The wild derivative, 04067–81–2–1–1 (B) (*C. echinospermum* interspecific line) grouped with desi S2 breeding lines, which had *C. echinospermum* in their pedigrees, whereas landrace ICC3996 grouped with desi genotypes (Figure 2B). Similar results were observed in the dendrogram generated using the maximum likelihood clustering method (Figure 2C and Supplementary Figure 1).

The LD pattern and LD-decay for all chromosomes (genome-wide) and each of the eight chickpea chromosomes (Ca1–Ca8) were estimated to gain insight into the extent of genetic diversity in the breeding germplasm. The decreasing trend of the LD decay curve with the increase in physical distance (Mbp) was observed based on a stochastic gamma model with an inverse link (Figure 2D). The LD decay at *r^2^* = 0.2, was 0.7 Mbp for the whole genome (average of all eight chromosomes). It varied among chromosomes and decayed at a faster rate in Ca4 (*r^2^* = 0.2; 0.38 Mbp) as compared with Ca6 (*r^2^* = 0.2; 3.26 Mbp) (Supplementary Figure 2), reflecting the variable recombination rates in the chickpea genome.

### Single-Locus and Multi-Locus Based Genome-Wide Association of Ascochyta Blight Resistance in Australian Chickpea Germplasm

**FIGURE 3:**
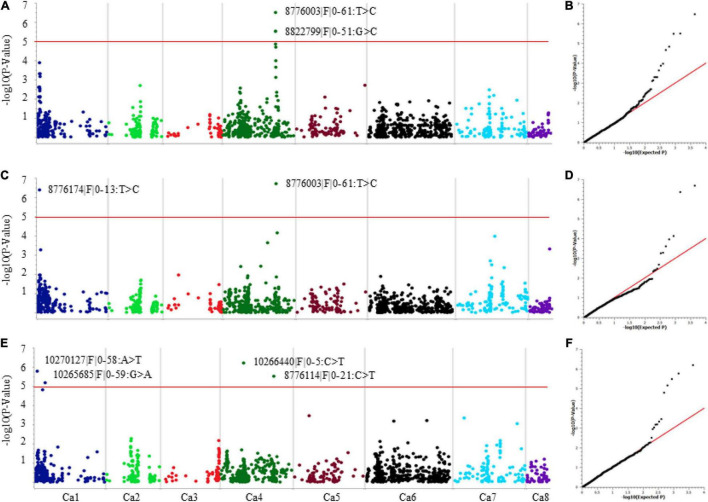
Manhattan and quantile–quantile (Q–Q) plots showing association of significant SNP with resistance to *A. rabiei* **(A,B)** Isolate F17191-1 using SLMM. **(C,D)** Isolate F17191-1 using MLMM. **(E,F)** Isolate TR9571 using MLMM. For Manhattan plots, the X-axis represents SNP positions across the eight chickpea chromosomes and the Y-axis is the negative logarithm *p*-value: –log10 (*p*) of each SNP. For Q–Q plots, X-axis represents expected –log10 (*p*) and Y-axis is observed –log10 (*p*) for each SNP.

Genome-wide association analysis was performed using two methods: single-locus mixed model (SLMM) and multi-locus mixed model (MLMM) while accounting for both genetic structure (PCA) and relatedness (identity-by-descent matrix) to reduce false positives and correct any spurious associations. The QQ-plots and Manhattan plots generated for SNP associations are presented in Figures 3A–F. A total of eight significant SNP associations (−log_10_ *P* values ≥ 5.0 and FDR ≤ 3.9E^–3^) were identified for AB resistance against F17191-1 and TR9571 isolates on chromosomes Ca1 and Ca4 (Table 2). A SNP on Ca4 (36231638 bp) was identified with both SLMM and MLMM approaches, while the MLMM approach identified an additional SNP on Ca1 (1820681 bp) for resistance against F17191-1 isolate (Table 2 and Figures 3A,C). Our results showed that different loci control resistance to *A. rabiei* isolates, as different genomic regions on Ca1 and Ca4 associated with AB resistance against F17191-1 and TR9571 were identified (Figures 3A,C,E). The proportion of the phenotypic variation (PV%) explained by SNP markers varied from 7.83 to 10.33% (Table 2).

### Haplotype Based Genome-Wide Association of Ascochyta Blight Resistance in Australian Chickpea Germplasm

We further used DArTseq and KASP SNP markers to estimate haplotypes and identified HB associated with AB resistance using HTR. A total of 215 HB were identified (Supplementary Table 3). The chromosome Ca4 had the greatest number of HB (60; 206 SNPs), compared with Ca8 (6 HB; 25 SNPs). The number of SNP markers in HB varied from two to 11 (Supplementary Table 3).

**FIGURE 4:**
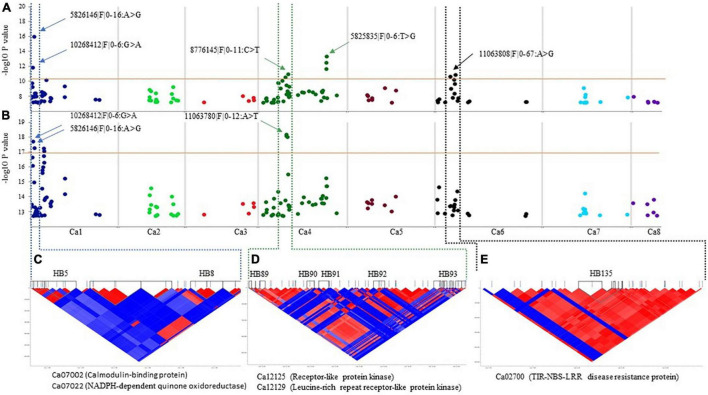
Manhattan plots showing association of the significant SNPs and corresponding pair–wise LD blocks on chickpea chromosomes using Haplotype Trend Regression. **(A)** SNP on Ca1, Ca4, and Ca6 associated with resistance to *A. rabiei* isolate F17191–1. **(B)** SNP on Ca1 and Ca4 associated with resistance to *A. rabiei* isolate TR9571. The dotted lines denote the regions that contain the significant SNP and the corresponding HB on Ca1 **(C)**, Ca4 **(D)**, and Ca6 **(E)**. Putative candidate genes underlying a genetic loci for AB resistance in the HBs are shown.

The HTR identified 21 HB on Ca1, Ca4, and Ca6 that were significantly associated with AB resistance, evaluated with both isolates (Table 3). Of these, ten HB were associated with resistance against F17191-1 isolate; two each on Ca1; HB5 (Ca1:992178–1108145 bp) and HB8 (Ca1:1886221–1976301 bp), Ca6; HB135 (Ca6:8125348–8262406) and HB141 (Ca6:11049083–11138183), and six on Ca4; HB89 (Ca4:14931208–15003392 bp), HB93 (Ca4:16478514–16635135 bp), HB110 (Ca4:37829979–37830058 bp), HB111 (Ca4:37856016–37991817 bp), HB112 (Ca4: 38027338–38123012 bp), and HB120 (Ca4:56597931–56643812 bp) (Table 3 and Figures 4A,C–E). All ten haplotypes explained moderate (19.95%) to high (27.96%) proportion of variation for resistance to F17191-1 isolate. In comparison, eleven HB for resistance against TR9571 isolate were identified on Ca1, and Ca4 and explained 29.52–32.68% of the phenotypic variation (Table 3 and Figures 4B–D). Noticeably, two HB; HB5 (Ca1: 992178–1108145 bp) and HB8 (Ca1: 1886221–1976301 bp) showed a significant association with resistance to both isolates and accounted for 22.95–31.10% of the phenotypic variation (Table 3). HTR analysis identified many genomic regions that were not detected with individual SNP-based SLMM and MLMM approaches and showed highly significant SNP associations (−log_10_ *P-value* = 10.43–19.15) for AB resistance, accounting for moderate to high phenotypic variation (19.95–25.39%, Table 3). Our results show that the haplotypes-based analysis can identify significant SNPs associated with AB resistance in chickpea germplasm against highly aggressive isolates.

**TABLE 3:** Summary of genetic loci associated with resistance against two *Ascochyta rabiei* isolates using Haplotype Trend Regression (HTR) in a GWAS set of Australian chickpea breeding genotypes.

Isolate	HB	Chromosome	Physical Position (bp)	First Marker in HB	Markers in HB	No of DArTseq markers in the HB	*P*-Value	Bonferroni *P*	FDR	PV (%)
F17191-1	5	Ca1	992178-1108145	10268412| F| 0-6:G > A	10268412| F| 0-6:G > A, 15990186| F| 0-51:G > C, 23870754| F| 0-8:G > T, 5825528| F| 0-21:A > C, 10267161| F| 0-22:C > T, 10268547| F| 0-33:G > A, 10265668| F| 0-61:C > G	7	11.74	5.19E-04	1.30E-04	22.95
	8	Ca1	1886221-1976301	5826146| F| 0-16:A > G	5826146| F| 0-16:A > G, 5825490| F| 0-61:A > T, 8776077| F| 0-27:G > A, 5825728| F| 0-33:C > T, 5825483| F| 0-12:A > G	5	15.90	1.65E-08	1.65E-08	27.96
	89	Ca4	14931208–15003392	8776080| F| 0-44:G > A	8776080| F| 0-44:G > A, 8776155| F| 0-45:G > T	2	10.43	1.01E-02	1.01E-03	19.95
	93	Ca4	16478514–16635135	8776145| F| 0-11:C > T	8776145| F| 0-11:C > T, 8776039| F| 0-48:C > A, 8776037| F| 0-68:A > T, 10268380| F| 0-58:C > T, 10266484| F| 0-36:C > G, 10265186| F| 0-65:C > T, 5826163| F| 0-14:C > T, 5825449| F| 0-22:C > T	8	10.88	4.11E-03	6.85E-04	21.63
	110	Ca4	37829979–37830058	11064111| F| 0-36:A > G	11064111| F| 0-36:A > G, 15979457| F| 0-60:C > T	2	11.56	6.11E-04	1.22E-04	21.68
	111	Ca4	37856016–37991817	5826025| F| 0-36:C > T	5826025| F| 0-36:C > T, 5825419| F| 0-36:A > G	2	12.40	1.09E-04	3.62E-05	23.93
	112	Ca4	38027338–38123012	5825835| F| 0-6:T > G	5825835| F| 0-6:T > G, 5826257| F| 0-23:T > C, 5825174| F| 0-55:G > C, 5825899| F| 0-27:A > G, 11063609| F| 0-24:C > T	5	13.24	1.46E-05	7.28E-06	25.17
	120	Ca4	56597931–56643812	10270073| F| 0-48:G > A	10270073| F| 0-48:G > A, 15990248| F| 0-13:C > A	2	10.48	8.92E-03	9.91E-04	20.03
	135	Ca6	8125348-8262406	11063808| F| 0-67:A > G	11063808| F| 0-67:A > G, 10264973| F| 0-46:A > G, 10266465| F| 0-45:A > G	3	10.56	8.87E-03	1.11E-03	21.14
	141	Ca6	11049083–11138183	10266288| F| 0-61:A > C	10266288| F| 0-61:A > C, 10266152| F| 0-13:C > T	2	10.77	4.37E-03	6.25E-04	20.47
TR9571	3	Ca1	753982–908551	10263753| F| 0-16:A > G	10263753| F| 0-16:A > G, 15990253| F| 0-37:G > A, 10264166| F| 0-49:T > C, 15990210| F| 0-55:T > A, 10263851| F| 0-65:C > T, 15990270| F| 0-53:A > G	6	17.62	3.05E-03	3.39E-04	30.29
	4	Ca1	924691–973310	10268610| F| 0-44:A > G	10268610| F| 0-44:A > G, 10264851| F| 0-12:G > C, 10266086| F| 0-36:T > A, 23870821| F| 0-31:A > T, 29631667| F| 0-9:G > A, 10263639| F| 0-27:C > G	6	17.05	1.25E-02	1.14E-03	29.52
	5	Ca1	992178–1108145	10268412| F| 0-6:G > A	10268412| F| 0-6:G > A, 15990186| F| 0-51:G > C, 23870754| F| 0-8:G > T, 5825528| F| 0-21:A > C, 10267161| F| 0-22:C > T, 10268547| F| 0-33:G > A, 10265668| F| 0-61:C > G	7	18.67	2.50E-04	6.24E-05	32.68
	8	Ca1	1886221–1976301	5826146| F| 0-16:A > G	5826146| F| 0-16:A > G, 5825490| F| 0-61:A > T, 8776077| F| 0-27:G > A, 5825728| F| 0-33:C > T, 5825483| F| 0-12:A > G	5	18.22	6.87E-04	1.37E-04	31.10
	29	Ca1	7007144–7167009	29967414| F| 0-9:T > G	29967414| F| 0-9:T > G, 10265744| F| 0-22:C > A, 10270116| F| 0-65:T > C	3	17.71	2.45E-03	3.07E-04	30.41
	30	Ca1	7168618–7169815	10267561| F| 0-20:A > G	10267561| F| 0-20:A > G, 10263699| F| 0-7:T > C	2	18.22	7.04E-04	1.17E-04	31.09
	31	Ca1	7275740–7285403	10270051| F| 0-40:T > C	10270051| F| 0-40:T > C, 10265685| F| 0-59:G > A	2	18.02	1.15E-03	1.64E-04	30.82
	32	Ca1	7306441–7456870	10263656| F| 0-64:C > T	10263656| F| 0-64:C > T, 10265001| F| 0-19:A > G, 10268478| F| 0-37:C > T, 5824850| F| 0-16:G > A	4	17.27	7.26E-03	7.26E-04	29.82
	90	Ca4	15413014–15448710	11063780| F| 0-12:A > T	11063780| F| 0-12:A > T, 15979307| F| 0-15:A > C, 5824854| F| 0-38:G > C	3	19.15	7.33E-05	7.33E-05	32.30
	91	Ca4	15484345–15562356	13146286| F| 0-51:A > C	13146286| F| 0-51:A > C, 15990257| F| 0-51:A > T	2	19.05	9.32E-05	4.66E-05	32.18
	92	Ca4	15977950–16026733	11063996| F| 0-5:T > A	11063996| F| 0-5:T > A, 10269593| F| 0-7:T > A, 10265886| F| 0-48:T > G, 10267389| F| 0-17:T > A, 11063189| F| 0-9:A > G	5	19.00	1.06E-04	3.53E-05	32.11

*HB, haplotype block; FDR, false discovery rate; PV, percentage of phenotypic variation; Physical position is based on Kabuli Reference genome; Frontier v2.6.3.*

### Verification of Genetic Linkage for Resistance in Biparental (F_3_) Populations

Two F_3_ populations derived from PBA Drummond/CICA1841 and CBA Captain/CICA1841 crosses were used to validate genomic regions associated with resistance to AB detected in the GWAS set. PC distribution of the GWAS set with PBA Drummond/CICA1841, and with CBA Captain /CICA1841 F_3_ populations suggests that both populations are related to the GWAS set (Supplementary Figures 3A,B).

Of the 1,432 polymorphic DArTseq SNP markers in Drummond/CICA1841 population, only 390 SNP markers with MAF frequency > 0.05 were used for linkage analysis using linear marker regression. In total, 17 genomic regions on Ca1 (1698056–349608 bp), Ca2 (17214916–18485579 bp), and Ca7 (2352759 bp and 23532379–23744945 bp) were significantly associated (−log10 *P*-Value > 4.5 and Bonferroni < 0.013) with resistance to TR9571 isolate (Supplementary Table 4).

For the CBA Captain /CICA1841 population, 31 genomic regions for resistance to TR9571 isolate were identified on chromosome Ca1. All the significant SNPs (31) were localized in a large genomic interval spanning 1057756–2545315 bp (Supplementary Table 5). Ca1 genomic region was associated with AB resistance in both F_3_ populations (Supplementary Tables 4, 5). This chromosomal region was also identified for resistance to F17191-1 and TR9571 isolates in the GWAS set using MLMM and haplotype-based GWAS approaches (Tables 2, 3 and Supplementary Tables 4, 5).

### Identification of Candidate Genes Underlying Quantitative Trait Loci

The putative candidate genes underlying sixty-one genomic loci anchored on the Ca1 (35), Ca2 (10), Ca4 (11), Ca6 (2), and Ca7 (3), identified using SNP and haplotype-based GWAS and in two F_3_ populations were searched against the CDC Frontier genome assembly v2.6.3 using BLASTn. Candidate genes that map within the 20 kb region up and downstream of the significant SNPs associated with resistance are presented (Table 2 and Supplementary Tables 4, 5, 7). Interestingly, 89 DArTseq SNPs that were significantly associated with resistance were localized within the annotated genes (Supplementary Table 8). For example, DArTseq SNP markers in HB8 (Ca1:1886221–1976301 bp) (significantly associated with both isolates), were located within the three candidate genes with known function in disease resistance; Ca07002 (calmodulin-binding protein; CaMBP), Ca07021 (NADPH–dependent quinone oxidoreductase) and Ca07022 (NADPH–dependent quinone oxidoreductase) (Supplementary Table 8 and Figure 4C). Another candidate gene, Ca02700 (TIR–NBS–LRR disease resistance protein) was identified in HB135 of chromosome Ca6 associated with resistance to F17191–1 isolate (Figure 4E). We also identified several candidate genes on chromosome Ca4, including protein kinase genes (Ca12125, Ca12129, and Ca12161) which are physically located near the SNP associated with resistance to TR9571 isolate (Figure 4D and Supplementary Table 7). Noticeably, we identified non-synonymous SNPs, causing amino acid changes in protein sequence of Ca07022 (exon 3, A > G, lysine to asparagine), and Ca02700 (exon 4, A > G, cysteine to arginine) suggesting that sequence variants of both genes are probably responsible for some of the variation in AB resistance among GWAS accessions (Supplementary Table 8).

## Discussion

Resistance to AB is one of the most important traits required for the long-term sustainability of chickpea production worldwide. Identification of SNPs/haplotypes associated with resistance would provide a valuable molecular tool to improve the efficiency of selection for achieving higher level of resistance to AB in new chickpea varieties. In addition, the significantly associated markers for AB resistance can facilitate the identification of functional genes and gene-based markers; a valuable toolkit for chickpea breeders aiming to reduce disease risk while improving adaptation and yield.

### Genetic Variation for Resistance to Ascochyta Blight

In this study, genetic analyses of advanced (GWAS set of 251 genotypes) and early generation breeding accessions of the Australian chickpea breeding program (PBA/CBA) (two F_3_ populations) were performed to determine association between SNP markers and AB resistance. Phenotypic expression of AB disease is difficult to quantify, as the fungus infects leaf and stem tissue, and pods and seeds, therefore resistance among GWAS accessions using four different methods of disease assessment was initially evaluated. All disease measures: disease score, main stem breakages, stem lesions and branches diseased showed moderate to high correlations. For GWAS, a standardized disease score (1–9 scale) used by the majority of the scientific community, was adopted. A small proportion of genotypes (4%) showed moderate resistance (disease scores of ≤3) to the two highly aggressive Australian isolates suggesting that useful variation exists within chickpea breeding germplasm and could be utilized for genetic analysis and resistance breeding purposes. Some of these sources of resistance are currently being used as parents for developing new cultivars resistant to highly aggressive *A. rabiei* isolates in CBA.

### Genome-Wide Association Identified Significant SNP Associations for Resistance to Ascochyta Blight

Although the genetic architecture of AB resistance has been reported in chickpea, a limited number of associated markers have been used in the breeding programs due to the complexity of the chickpea × *A. rabiei* × environment interaction and resistance breakdown. The non-static nature of the pathogen populations due to high evolution potential and selection pressure posed by environmental factors and farming practices, (Ford et al., 2021) and the breeding germplasm, pose an on-going challenge for resistance breeding.

We utilized single and multi-locus (SLMM and MLMM) and haplotype-based (HTR) approaches to identify genome-wide SNP associations for resistance to AB in the Australian breeding germplasm (Tables 2, 3). The haplotype-based approach provides improved statistical power compared to the single SNP-based GWAS for identifying genetic loci for complex traits controlled by multiple genes or QTLs (Pei et al., 2009; Minamikawa et al., 2018). Furthermore, haplotype-based approach enables the detection of multiple alleles for marker-assisted breeding (Maldonado et al., 2019).

Our analysis detected a higher number of significant SNP associations using HTR compared to SLMM and MLMM thus providing more detailed marker-trait association. These results are in agreement with previous studies, which demonstrated that haplotype analysis captures associations, which are not detected using single-SNP based approaches as haplotypes are dependent on the mutational and recombinational history of the QTL and the near-by markers (Pei et al., 2009; Lorenz et al., 2010; Minamikawa et al., 2018; Maldonado et al., 2019). Haplotype-based SNP association also explained a higher proportion of the phenotypic variation (11.41–25.39%), compared with the single-SNP (3.29–10.38%) based analysis using SLMM/MLMM (Tables 2, 3), confirming the reliability of the HTR approach in detecting loci with a substantial contribution of phenotypic variation. The haplotype based approach has been employed in detecting trait-marker associations in barley, soybean, and maize (Lorenz et al., 2010; Contreras-Soto et al., 2017; Maldonado et al., 2019; Longmei et al., 2021), however, to date this approach has not been reported in chickpea.

Importantly, by combining single, multi-locus, and haplotype-based GWAS, we identified “hotspot” regions on chromosomes Ca1 and Ca4 associated with resistance to AB in chickpea. Genetic analyses of CBA Captain/CICA1841 and PBA Drummond/CICA1841 F_3_ populations revealed a large genomic region on Ca1 (1.10–3.5 Mbp) that was also detected by MLMM (Ca1: 1781336) and HTR (HB8; Ca1:1886221–1976301) (Tables 2, 3 and Supplementary Tables 4, 5). The Ca1 genomic region also corresponds to a previously identified QTL (CPR01–Qab1.1, PV = 11%, Ca1:1999318–5345307 bp) in ICCV96029/CDC Frontier population (Deokar et al., 2019a, see Supplementary Table 6). These findings confirm that genomic regions for resistance to *A. rabiei* can be tracked in Australian chickpea breeding germplasm and it is likely that some common genomic regions are associated with AB resistance in the Canadian cultivar, Frontier and the Australian chickpea germplasm.

We identified 325 candidate genes mapped within 20 kb up– and downstream of the SNP markers on Ca1, Ca2, Ca4, Ca6, and Ca7 and at least 70 candidate genes (89 SNPs) had the associated SNP within the gene sequence. The most significant genomic region on Ca1 associated with resistance to both isolates’ harbors three genes; Ca07002 (calmodulin–binding protein; CaMBP), and Ca07021 and Ca07022 (NADPH–dependent quinone oxidoreductase). In Arabidopsis, CaMBP may play a critical role in the cross-talk of multiple signaling pathways in the plant (Lv et al., 2019). Quinone oxidoreductases (QRs) are flavoproteins that are involved in NADPH oxidation-reduction process and protect organisms from oxidative stress in response to infection by necrotrophic fungi (Heyno et al., 2013). The role of CaMBP and QRs have not been reported in chickpea-*A. rabiei* interaction. Our results provide a basis to further investigate molecular mechanisms and associated gene pathways involved in a complex and an important pathosystem.

Genomic regions on Ca2 associated with resistance to TR9571 isolate identified in the PBA Drummond/CICA1841 F_3_ population (Ca2: 17214916–18485579) are within the QTL intervals which have been previously identified in the mapping populations derived from ICCV96029 × Amit and ICCV96029 /CDC-Luna (Anbessa et al., 2009; Deokar et al., 2019a,b). Nine candidate genes were identified in the proximity of the Ca2 genomic region and of these two genes, Ca29963 (inactive receptor-like protein kinase), and Ca29960 (ankyrin repeat) were located within 7 Kb of the DArTseq SNP markers in the F_3_ population derived from PBA Drummond/CICA1841. Deokar et al. (2019b) identified ABA receptor gene (ABA–R:17.3 Mbp) in the QTL interval qAB2.1 which coincides with the Ca2 genomic region associated with AB resistance in the F_3_ population derived from PBA Drummond/CICA1841. Moreover, two TIR-NBS-LRR (TNL) genes, LOC101513119 (18.2 Mbp) and LOC101493700 (18.3 Mbp) map within the Ca2 genomic region (Sagi et al., 2017).

On chromosome Ca4, nine HB were identified, consisting of a large genomic region (14.9–56.6 Mbp) which showed association with resistance to AB. These results corroborate with previous findings suggesting that chromosome Ca4 is an AB QTL hotspot region (Udupa and Baum, 2003; Iruela et al., 2006; Tar’an et al., 2007; Aryamanesh et al., 2010; Sabbavarapu et al., 2013; Daba et al., 2016; Kumar K. et al., 2018; Deokar et al., 2019a,b; Garg et al., 2019). Li et al. (2017) and Sudheesh et al. (2021) also identified a significant genomic region on Ca4 (15 to 16 Mbp; annotated in version 1 of the Kabuli genome), associated with AB resistance and reported 12 and 99 candidate genes, respectively. This genomic region corresponds to 14.52–15.56 Mbp of the Version 2.6.3 of the kabuli genome which is significantly associated with AB resistance (HB90) against isolate TR9571 in the GWAS set. We identified five candidate genes in this genomic region; Ca12123 (chaperone DnaJ domain protein), Ca12125 (probable receptor-like protein kinase), Ca12127 (uncharacterized protein), Ca12128 (probable serine/threonine-protein kinase), and Ca12129 (LRR receptor-like kinase gene), which correspond to Ca_05512, Ca_05516, Ca_05518, Ca_05520, and Ca_05515, respectively (kabuli genome v 1). The genomic region on Ca6 (HB135: 8125348-8262406) associated with AB resistance in our GWAS population overlaps with the AB-QTL previously reported by Sabbavarapu et al. (2013). A NBS-LRR-TNL (LOC101498642) identified in this region by Sagi et al. (2017) corresponds to the candidate gene Ca02700 (Ca6: 8224635-8228364) identified in our study. Further functional studies are required to identify causal genes in the genomic regions identified in our study and develop gene-based markers.

In summary, 26 genomic regions associated with resistance to AB in a GWAS set were identified and at least seven SNP associations were traced in CBA Captain/CICA1841 F_3_ population. Eighty-nine significantly associated SNPs with resistance to AB were located within the candidate genes; some of these genes are implicated in disease resistance in plants. The role of these candidate genes needs to be established to understand chickpea-Ascochyta interactions and develop strategies to enhance resistance in commercial cultivars. Both common and different SNP loci identified in this study offer the opportunity to combine the alleles associated with resistance to different isolates (genetic clustering and pathogenicity) to develop cultivars with “broad-spectrum” resistance to multiple *A. rabiei* isolates. It remains to be validated if the same or different genomic regions are associated with resistance/susceptibility to isolates based on their genetic clustering and pathogenicity grouping.

## Data Availability Statement

The original contributions presented in the study are included in the article/Supplementary Material, further inquiries can be directed to the corresponding author.

## Author Contributions

RR and KH planned the study and designed the experiments. AW, RR, and ND phenotyped the F3 populations. JD and MK-K phenotyped the GWAS set. RR and MR analyzed the data. KM developed the disease score system. NS and RR identified the candidate genes. RR performed GWAS analysis and wrote the manuscript. All authors read and approved this manuscript for publication.

## Conflict of Interest

The authors declare that the research was conducted in the absence of any commercial or financial relationships that could be construed as a potential conflict of interest.

## Publisher’s Note

All claims expressed in this article are solely those of the authors and do not necessarily represent those of their affiliated organizations, or those of the publisher, the editors and the reviewers. Any product that may be evaluated in this article, or claim that may be made by its manufacturer, is not guaranteed or endorsed by the publisher.

## Footnotes

1
http://www.cicer.info/cgi-bin/gb2/gbrowse/kabuliV2.6.3/

2
www.goldenhelix.com

## Supplementary Material

Supplementary Figure 1 A neighbor-joining phylogenetic tree of GWAS set of Australian chickpea genotypes.

Supplementary Figure 2 LD decay plots of all eight chickpea chromosomes and Ca1–Ca8. The squared correlation coefficient *r2* values (Y-axis) were plotted against the physical distance in mega base pairs (Mbp) (X-axis).

Supplementary Figure 3 Principal component analyses of GWAS set and F_3_ populations. **(A)** GWAS set and PBA Drummond/CICA1841 (based on 316 common DArTseq markers). **(B)** GWAS set and PBA Captain/CICA1841 (based on 272 common DArTseq markers); blue: kabuli genotypes, green: desi genotypes, light green: CICA1841, purple: PBA Drummond, cyan: CBA Captain, red: *C. echinospermum* interspecific line, maroon: landrace (ICC3996).
