# Supplementary figures and images for: Genome-Wide Association Analyses Track Genomic Regions for Resistance to Ascochyta rabiei in Australian Chickpea Breeding Germplasm
Source: Front Plant Sci. 2022 May 18;13:877266. doi: 10.3389/fpls.2022.877266 (PMC9159299; doi:10.3389/fpls.2022.877266)

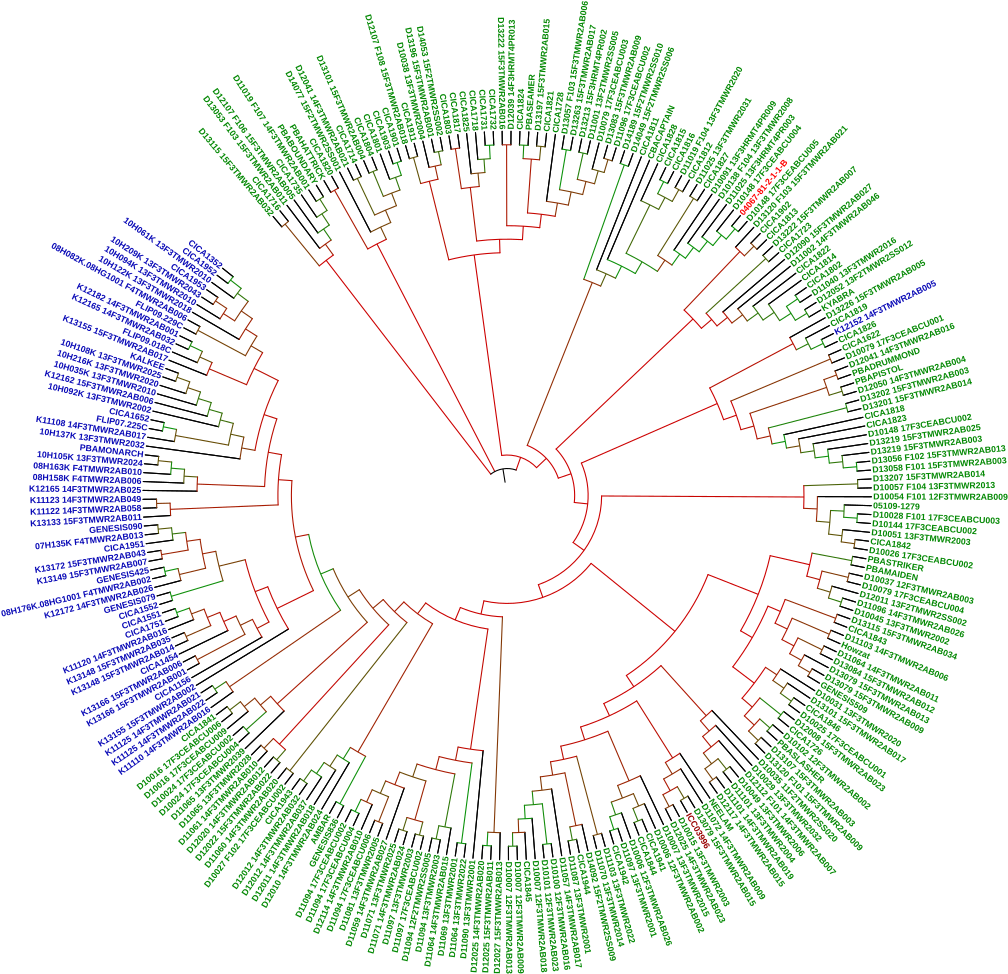


Supplementary Figure 1.

Supplement: Supplementary Figure 1 — A neighbor-joining phylogenetic tree of GWAS set of Australian chickpea genotypes. [file Table_10.DOCX]

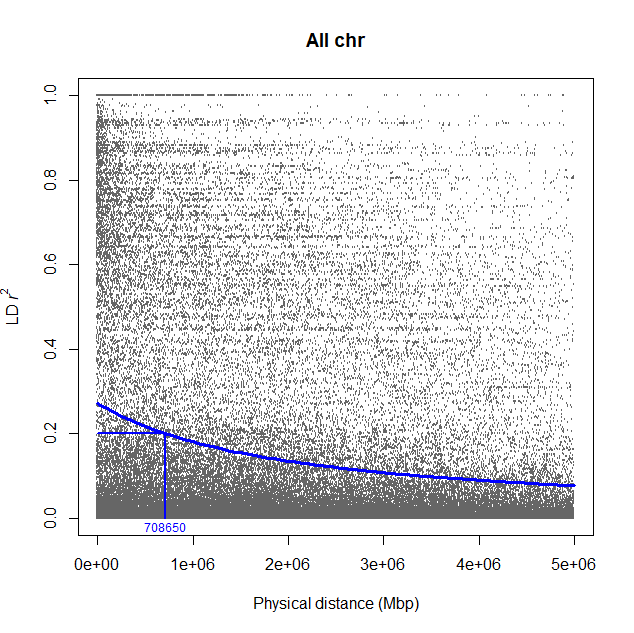

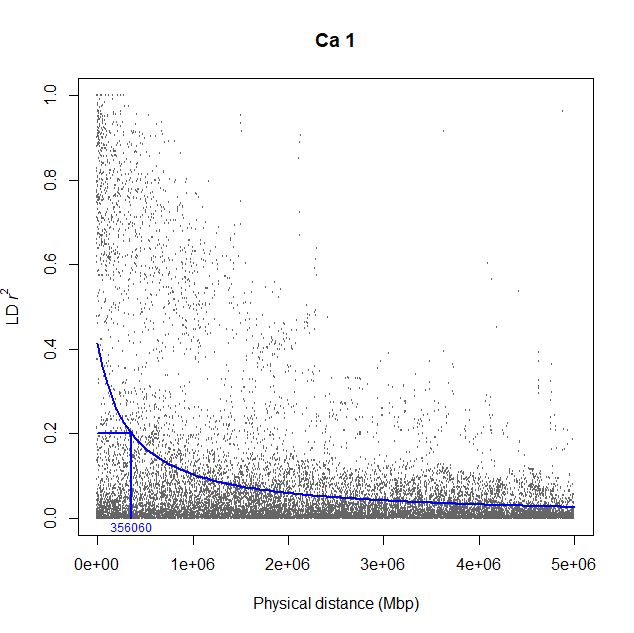

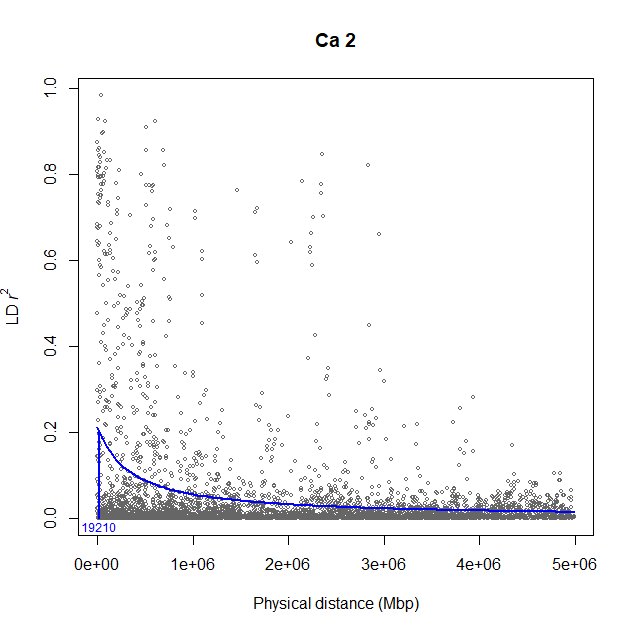


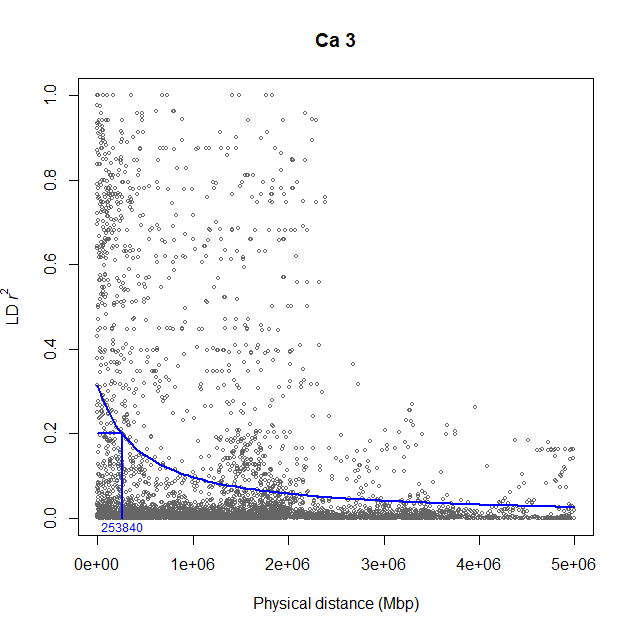

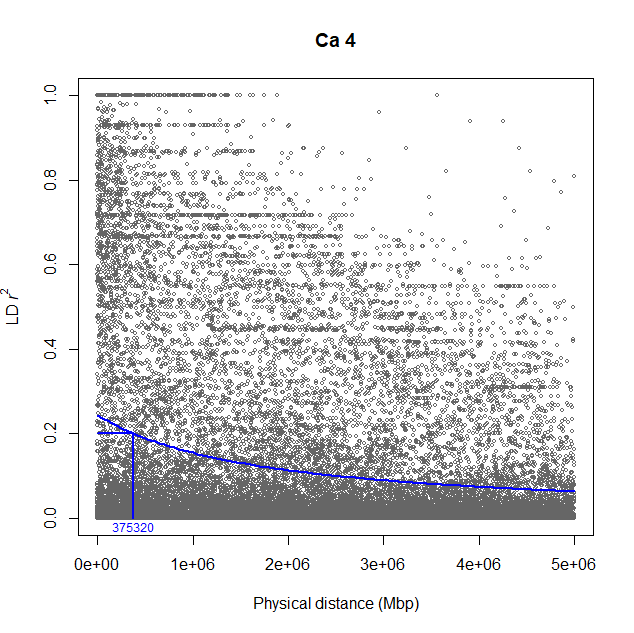

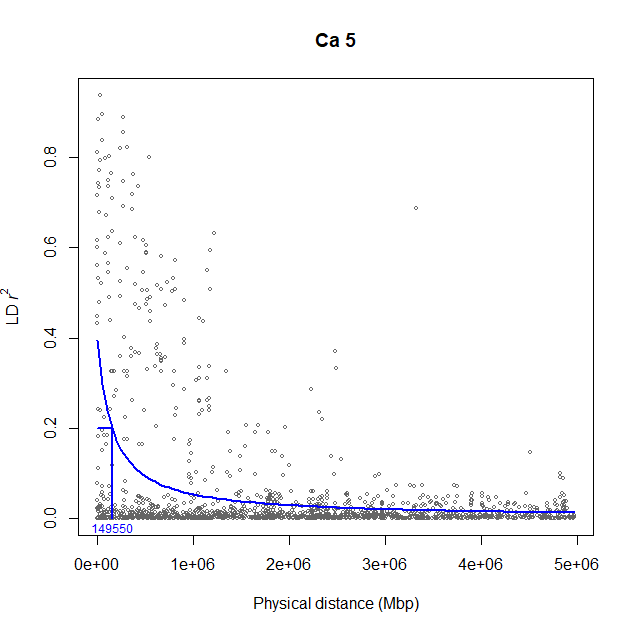


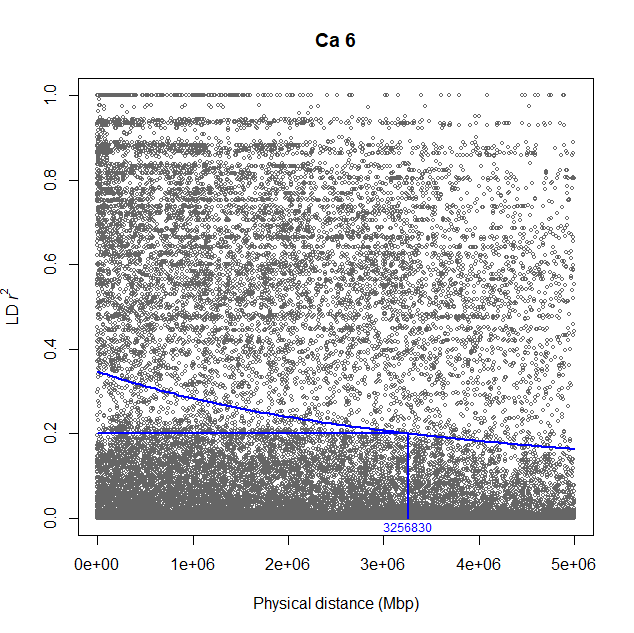

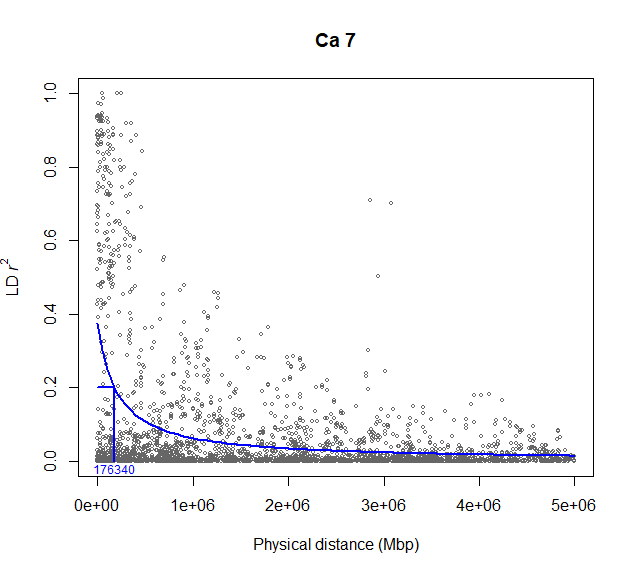

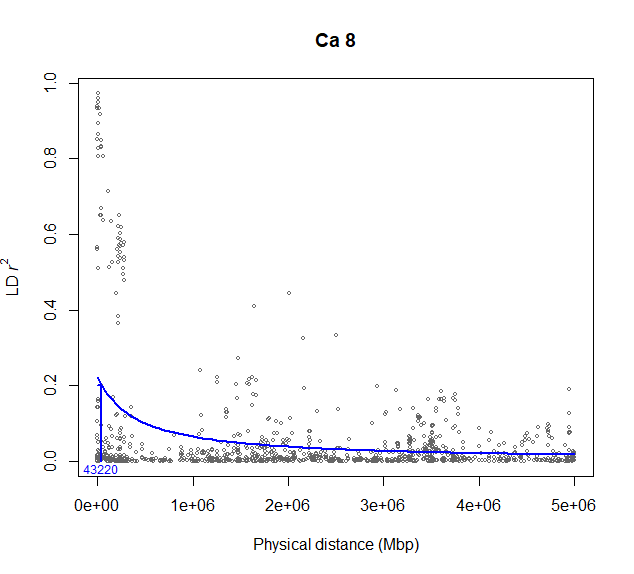


Supplementary Figure 2.

Supplement: Supplementary Figure 2 — LD decay plots of all eight chickpea chromosomes and Ca1–Ca8. The squared correlation coefficient r2 values (Y-axis) were plotted against the physical distance in mega base pairs (Mbp) (X-axis). [file Table_11.DOCX]

Supplementary Figure 3


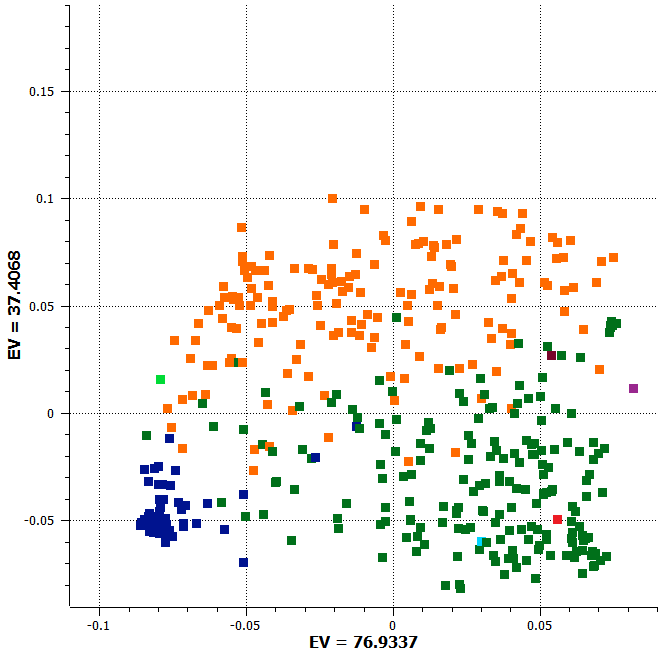

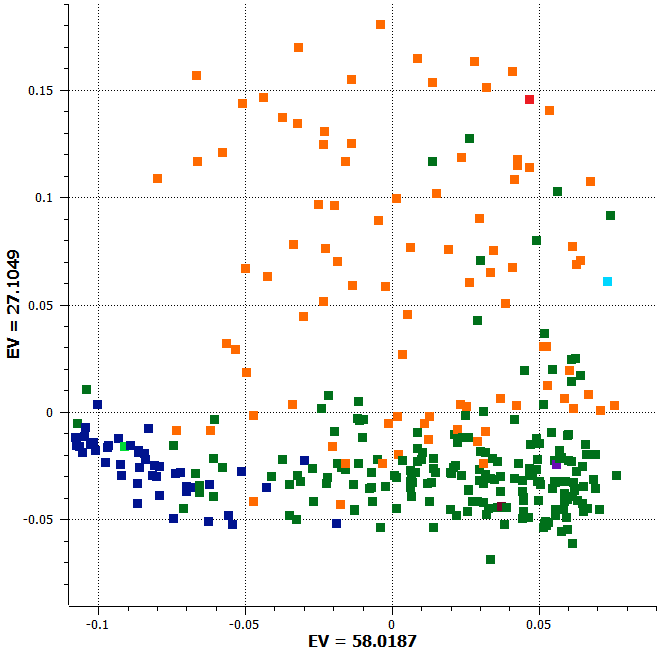


A

B

Supplement: Supplementary Figure 3 — Principal component analyses of GWAS set and F3 populations. (A) GWAS set and PBA Drummond/CICA1841 (based on 316 common DArTseq markers). (B) GWAS set and PBA Captain/CICA1841 (based on 272 common DArTseq markers); blue: kabuli genotypes, green: desi genotypes, light green: CICA1841, purple: PBA Drummond, cyan: CBA Captain, red: C. echinospermum interspecific line, maroon: landrace (ICC3996). [file Table_9.DOCX]
